# Poisoning by Vapors of Antimony

**Published:** 1842-03

**Authors:** 


*Poisoning by the Vapors of Antimony.*—M. Lohmeier, of Schonebeck, has recorded in the Nos. of *Casper's Wochenschrift* for April and May 1840, the history of four patients who were frequently exposed to the vapors of antimony, in an establishment where there were prepared on the large scale tartrate of antimony, butter and glass of antimony, and other antimonial preparations, during the preparation of which were disengaged abundant vapors of antimonious and antimonic acid, and chloride of antimony.

The four patients presented the following symptoms. Pain in the head; lancinating pain along the edge of the ribs and in the back; difficult respiration with mucous and sibilous rattles over the chest; difficult expectoration of tenacious mucus; sleeplessness, anorexia, diarrhœa, profuse perspirations, and general weakness; dysuria with a mucous discharge, causing a burning feeling in the urethra; flaccidity of the penis, with loss of the sexual appetite, and even complete impotence; pain in the testicles, and atrophy of these organs, as well as of the penis; pustules over different parts of the body, but especially on the thighs and scrotum.

M. Lohmeier observes, that in none of the recorded cases of poisoning by antimony, has the peculiar affection of the medicines on the sexual organs been discovered, nor yet its effect in exciting a peculiar cutaneous eruption; but it is remarked that the homœopathists have noticed this on the generative organs, and that its preparations are habitually administered to animals by agriculturalists to hasten their fattening, when it probably acts by diminishing the sexual appetite; and lastly, that it appears to have been used by monks in the monasteries with the view of diminishing their sexual propensities, and hence its name of antimony, from *moine*, a monk.

The curative means used by Mr. Lohmeier were the free use of antiphlogistics in the first place, and afterwards the internal administration of opium, tannin, and quinine, with lotions of the same applied to the parts externally affected, and milk diet. If the functions of the sexual organs did not return naturally as the other affections abated, he recommended the use of tincture of cantharides united with opiates, and lotions of cold water to the scrotum.—*B. and F. Med. Rev.*, Oct. 1840, and *Edin. Med. and Surg. Journ.* Jan. 1841.

